# Resolving the context-dependency of local heterogeneity–diversity relationships across rocky reefs worldwide

**DOI:** 10.1098/rspb.2024.2723

**Published:** 2025-05-07

**Authors:** Jordi Sola, John N. Griffin

**Affiliations:** ^1^ Department of Biosciences, Swansea University, Swansea SA2 8PP, UK

**Keywords:** biodiversity, habitat complexity, habitat structure, ecosystem functioning, topography, marine reefs

## Abstract

Environmental heterogeneity is widely thought to promote biodiversity, yet its variable effects limit its predictive power. This variability can be better understood by considering key mediating factors: different forms of local heterogeneity, organismal groups and their associated traits, and the broader environmental context (e.g. latitude). To address these factors, we analysed 144 studies (24 412 data points and 2815 effects) from rocky reefs worldwide. Heterogeneity was defined as spatial variability, and heterogeneity metrics were grouped into facets such as three-dimensional (3D) structure (e.g. substrate rugosity), complexity (e.g. fractal dimension) and feature variability (e.g. mussel size classes). All facets promoted biodiversity, but effects were context-dependent, with 3D structures having the strongest impact, likely owing to increased niche provision and substrate area. Responses also varied across organismal groups, with small-bodied and mobile species benefiting the most. Additionally, heterogeneity reduced grazing and enhanced recruitment, helping promote biodiversity. Effects were strongest on biogenic substrates, at lower latitudes and in more stressful intertidal zones. Overall, the influence of local heterogeneity depends on how it is generated and measured, organisms’ traits, and prevailing environmental conditions. These insights inform the development of a conceptual model predicting heterogeneity’s context-dependent effects on biodiversity across systems.

## 1. Introduction

The role of environmental heterogeneity in structuring ecological communities remains a focal and debated topic within ecology [1–3]. Spatial heterogeneity, integral to ecosystem complexity, represents the variability of environmental conditions or resources across spatial scales—from local to landscape and regional levels [4]. This heterogeneity, shaped by forces such as climate, geology and habitat-forming species [5], is an important consideration in conservation and restoration strategies aiming to support biodiversity and ecological resilience. At local scales, where individual organisms interact, heterogeneity generated by abiotic (e.g. substrate topography) and biotic or biogenic (e.g. canopy-forming species) elements mediates species interactions with one another and with their microhabitats, significantly influencing community structure, diversity and processes such as grazing and recruitment [5–7]. Yet, the specific nature of heterogeneity–diversity relationships (HDRs) remains contested [8]. Synthesis of HDRs from well studied systems helps assess sources of variability in these fundamental relationships and identify key factors that consistently moderate heterogeneity’s effects. Such insights may further aid conservation and restoration practitioners in leveraging heterogeneity to promote biodiversity and ecosystem function in management and recovery plans.

Current uncertainty in local HDRs stems from differing theoretical expectations and variability among empirical findings. Classically, local heterogeneity has been thought to promote diversity through increases in niche availability [9,10], or by enhancing productivity potential [11,12]. Additional mechanisms generating HDRs include heterogeneity-provisioned resources, and refugia from physical stress or biological interactions [13]. Furthermore, widespread sources of heterogeneity, such as topography (rugosity) or structure-forming species, also increase realized (a)biotic substrate area, even when the planar area is held constant, introducing additional pathways through which heterogeneous environments can encourage diversity. However, other recent theory argues that highly heterogeneous environments excessively divide individual habitats and reduce diversity [14] and the presence of specialist species [15]. Moreover, while positive HDRs are often assumed [10], weak or negative effects are also common [8,16], further questioning their universality.

Identifying patterns in HDRs is crucial for understanding heterogeneity’s ecological roles across systems. To do so requires identifying key factors that influence HDR variability. As heterogeneity often provides refugia from environmental stressors, which would otherwise reduce diversity owing to increased trait filtering [17–19], HDRs may become more pronounced under extreme conditions, such as in intertidal zones, where elevated desiccation stress intensifies environmental filtering outside refugia [20]. Organismal traits, like body size and locomotion, also influence the extent to which certain species benefit from small-scale heterogeneity, such as microtopographic variations or complex canopy structures [21]. Some effects may further be indirect, mediated through ecological processes like grazing [22], predation [23] and recruitment [24]. For instance, marine artificial structures and hedgerows in agricultural systems have been shown to promote species diversity by enhancing recruitment [25,26]. Local heterogeneity has also been used to explain biodiversity, ecosystem services (e.g. fisheries productivity, carbon sequestration) and resilience to environmental stressors [25,27–31]; but the observed variability in heterogeneity’s effects and mechanisms across ecological contexts remains largely unexplored. Synthesizing HDRs established across various contexts may help identify key factors and lead to a more comprehensive understanding of these relationships.

Nonetheless, synthesis efforts continue to be hampered by the various ways that heterogeneity is generated, quantified and conceptualized (>100 potential terms and metrics; [25,26]). Heterogeneity is generated and measured in many ways, such as through structural features of varied sizes (e.g. small versus large crevices), or in two (2D) (e.g. tree cover) versus three dimensions (3D) (e.g. tree height across a plot) [1]. Heterogeneity can also encompass additional concepts, such as structural complexity, as measured by fractal dimension [32]. Such heterogeneity forms, or facets, represent distinct aspects of spatial variation, considering both 2D and 3D variations of the substrate and variations in substrate amount and its features. These facets influence the distribution of refugia and resources, which likely have consequences for organism–environment interactions and diversity responses [1]. For example, the size of features such as crevices determines which organisms can access these environments [33], while vertical structures such as tree canopies create refugia from ground-dwelling predators and often greatly increase local surface area, enhancing settlement substrate and resource availability [34]. Thus, synthesis efforts need a framework that organizes metrics into distinguishable forms of heterogeneity, avoiding mixing different forms of heterogeneity that potentially have distinct influences on communities.

Rocky reefs provide an archetypal system to apply such a framework and contribute towards the general understanding of local heterogeneity effects on ecological communities. Rocky reefs are distributed along coastlines worldwide. They span from the relatively environmentally stressful intertidal (e.g. thermal stress [35]) to the less stressful subtidal environments, and provide key services such as coastal protection, fisheries provision and carbon sequestration [36]. Physical structures generating heterogeneity within reefs are highly varied and translatable to other systems, such as canopy (e.g. kelp canopy), topographic (e.g. rockpools) and microscale features (e.g. rock rugosity or topography at millimetre scale or smaller), and influence variability in environmental conditions such as water flow and temperature [37]. Since rocky reef studies have been conducted considering multiple facets of heterogeneity, organismal groups and various environmental contexts (e.g. subtidal versus intertidal, tropical versus temperate, rock versus biogenic substrates), this body of literature provides a valuable opportunity to improve our understanding of HDRs and key sources of context-dependency.

Here, we synthesize HDRs for rocky reef communities worldwide across explicitly defined heterogeneity facets. Our principal aim is to identify key factors explaining context-dependency in HDRs. Beyond diversity, we further aim to determine how heterogeneity influences other related properties and processes, including the abundance and biomass of organisms, and the rates of ecological processes such as recruitment and grazing. To address context-dependency and the broader influences of heterogeneity, our objectives are to: (i) evaluate and compare the strength and form of heterogeneity effects on community responses across facets (a), organismal groups (b) and ecological processes (e.g. grazing, recruitment) (c); and (ii) identify key biogeographical and environmental factors that contextualize the variation in HDRs.

First, we hypothesize that HDRs will strengthen in response to facets that represent 3D habitat variability since at local scales the increase in 3D habitat will provide the largest increases of surface area as well as indirectly increase variability in environmental conditions. Second, areas with greater environmental stress will heighten the importance of heterogeneity-driven amelioration, as the refugia provided by heterogeneity will be more beneficial—and fewer species and organisms will survive outside them. Third, heterogeneity will benefit smaller and more vertically mobile animal groups the most, as they are better able to capitalize on small-scale and 3D environmental variation. Combined, the results will reveal key factors explaining the variability among HDRs on rocky reefs, allowing us to develop hypotheses for testing the context-dependency of heterogeneity effects across systems.

## 2. Methods

### (a) Study inclusion

We conducted a comprehensive literature search on Web of Science from July 2020 to June 2021, focusing on observational and experimental studies published between 1950 and 2020. The search string combined terms related to habitat heterogeneity [37], diversity indices and ecological processes such as productivity, grazing or recruitment, with terms specific to subtidal and intertidal rocky reefs or shores, excluding other systems like mudflats (full-search string in electronic supplementary material, S1). After removing duplicates, we obtained 6192 studies. We then excluded additional studies that did not focus on rocky reefs (e.g. centred on artificial reefs), lacked observational or experimental data, failed to assess heterogeneity effects or did not address diversity or ecological processes. Studies without a clear *a priori* aim to investigate heterogeneity effects or those exclusively examining habitat fragmentation were also excluded.

To avoid confounding heterogeneity with plot surface area, we removed studies that varied plot size within an HDR. However, we retained studies measuring variable 3D surface area within plots of standardized basal area. For example, studies assessing crevice or canopy effects were included if they adhered to fixed plot sizes, as this realized surface area of available substrate (e.g. fronds of canopy-forming seaweed) often varies with heterogeneity. We reviewed the remaining studies for sample size, the number of heterogeneity levels considered, and experimental or survey methods to identify design attributes such as the use of appropriate response variables (full inclusion criteria and PRISMA workflow in electronic supplementary material, S2). Studies with identified design weaknesses (e.g. confounding heterogeneity with site or date) were included in sensitivity analyses to assess their effect on the model outputs. In total, 144 studies, representing 2815 individual relationships and 24 412 data points, met our inclusion criteria (overview on geographical and data distribution in electronic supplementary material, S3).

### (b) Data extraction and definition of heterogeneity facets

For each study, we gathered data on location, methodology, heterogeneity measurement, taxon, community response and ecological process metrics (electronic supplementary material, S4). Community response variables included diversity metrics (richness, evenness and Shannon index), abundance and biomass. Ecological process metrics encompassed those used to quantify grazing, predation, recruitment and body size. Body size was incorporated as an indirect proxy for ecological processes owing to its well documented ecological significance [38]. To avoid confounding by site, time or substrate, data within each study were aggregated into HDR groups sharing these factors. In cases where fewer than three replicates were available within a given site, date or substrate (19% of studies), assessments were extended across different sites, dates or substrates, following the approach used by the original authors.

**Figure 1.**
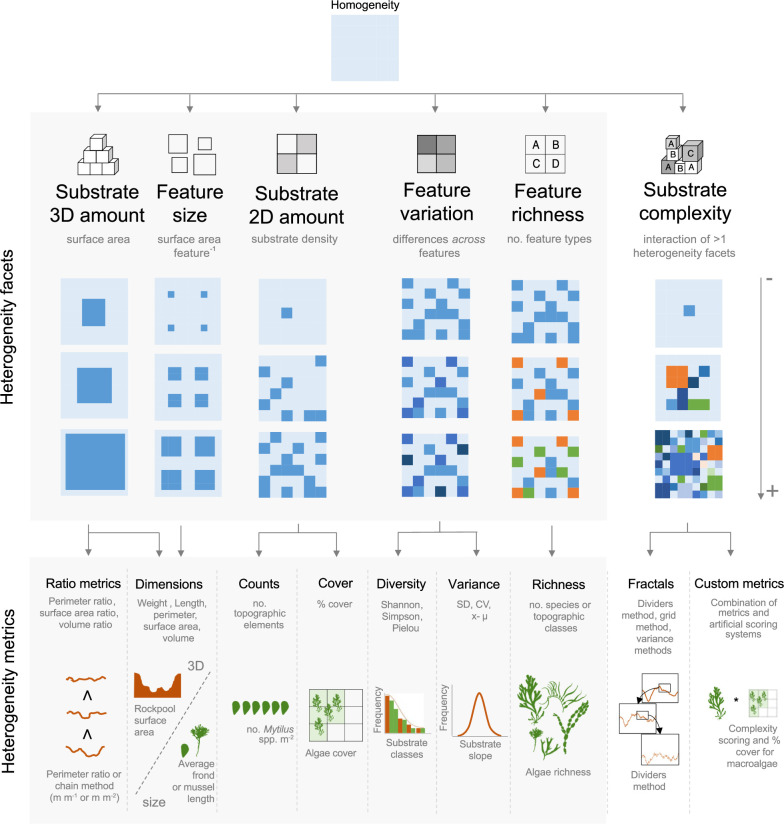
Conceptual overview of heterogeneity facets and metrics in rocky reef ecosystems. This figure presents the conceptual and graphical definitions of heterogeneity facets, capturing the complexity of spatial variation in rocky reef environments and linking each facet to the metrics in the literature. These facets reflect the total amount of substrate (substrate 3D and 2D amounts), the characteristics or features of the substrate (feature size, feature variation, feature richness), and the interactions across facets (substrate complexity). These categories reflect both structural and compositional dimensions of heterogeneity. Note that a brief definition of each facet is provided beneath its respective symbol. For example, feature size refers to surface area per feature (e.g. macroalga or rock crevice). Feature variation reflects differences observed across features, such as variation in species composition or topographic features such as substrate classes (e.g., measured by Shannon diversity). It can also capture quantitative differences, such as topographic variability expressed through metrics like Standard Deviation (SD), Coefficient of Variation (CV), or deviations from the mean (x – µ). Additional metric details are provided in electronic supplementary material, S5.

Heterogeneity metrics across studies were grouped into general categories referred to as heterogeneity facets, which represent distinct forms of spatial variation in the environment (figure 1). These facets capture both vertical and horizontal variation in physical substrates, as well as variations in the identity, diversity or distribution of physical features (electronic supplementary material, S5). To account for the full range of spatial heterogeneity in a plot, we defined six heterogeneity facets based on established metrics in the literature [1,4,32,39]. These facets were: (i) substrate 3D amount—the total surface area of 3D substrates within a fixed basal plot area, with e.g. volume or biomass as proxies [40]; (ii) feature size—the average surface area, volume, height or biomass of features like holes, fronds or mussels [40]; (iii) substrate 2D amount—the relative surface area or density of features within a 2D plot [41]; (iv) feature variation—the variability in feature size, volume, biomass or identity within a plot [42]; (v) feature richness—the number of distinct feature types, such as species or structural classes [43]; and (vi) substrate complexity—the interaction between two or more facets, measured through combined metrics, author-defined treatments or fractal indices [4,32,44].

### (c) Transforming heterogeneity data

For each HDR group, we *z*-transformed heterogeneity data and then converted it to the 0−1 range. For studies with discrete heterogeneity descriptors, we similarly created a variable on a 0−1 scale, adjusting the levels (e.g. 0 and 1; 0, 0.5 and 1) according to the study’s classifications (e.g. present versus absent, low versus medium versus high). This standardization accounted for differences in heterogeneity metrics and the magnitude of heterogeneity changes across studies. To further control for these factors, we calculated the coefficient of variation (CV) for the raw heterogeneity data in each HDR, based on the original units.

### (d) Calculating effect sizes

Effect sizes were quantified per observation using the natural logarithmic response ratio (lnRR), which provides a measure of the proportional change in response variables between treatments.


$${lnRR}_{i}=ln\left(\frac{y_{i}}{mean\left(min\left(y\right)\right)}\right),$$

where *y_i_* represents the response value for a given heterogeneity level and mean(min(*y*)) is the mean response value at the lowest heterogeneity level in each HDR [45]. For studies using discrete heterogeneity descriptors, we adapted this formula to compare the response values with the mean response from the treatment with the lowest heterogeneity. This approach allowed consistent comparisons across studies with varying experimental designs.

### (e) Data analysis

We used linear mixed-effects models to analyse HDRs, incorporating multiple covariates to account for variation. Heterogeneity was modelled as a fixed quadratic term (electronic supplementary material, S6), with heterogeneity CV added as an interacting covariate when significant, to capture differences in heterogeneity gradients. Random effects included study ID and plot ID, accounting for variation in HDR slopes and intercepts across studies [46,47]. Additional fixed effects and interactions were selected based on *a priori* expectations (see §§ 2f,g), with non-significant interactions excluded from the final models [48]. To avoid ecological biases and unrealistic extrapolations, we modelled heterogeneity patterns separately across different facets, organismal groups and ecological processes. A detailed assessment of the data confirmed this approach yielded more accurate and ecologically meaningful results (electronic supplementary material, S7–S9 and S13).

### (f) Heterogeneity effects across facets, organismal groups and ecological processes (objective 1)

To evaluate heterogeneity effects across facets, we aggregated data from microinvertebrate and macroinvertebrate organismal groups, ensuring all facets were represented for species richness models and abundance models (electronic supplementary material, S7 and S8). Additional covariates included the number of species per HDR (abundance models) and organismal group (both species richness and abundance models).

We then assessed heterogeneity effects across organismal groups by running a separate model per group. Each organismal group represented a common category used in rocky reef literature: primary producers (micro- and macroalgae), invertebrates (microinvertebrates, macroinvertebrates and large macroinvertebrates) and fish. These groups differ in key traits such as mobility and size, which influence their capacity to exploit heterogeneous environments (electronic supplementary material, S8). In each model, we analysed multiple community response metrics (abundance, biomass, diversity and richness), pooled as the response variable. Alongside the covariates and random effects described above, we added community response metric type as a fixed effect, interacting with both heterogeneity and heterogeneity facet. Owing to information on multiple facets missing for some organismal groups, we grouped data on substrate complexity, feature size, feature variation and feature richness under ‘other heterogeneity facets’.

Finally, we evaluated heterogeneity effects on four ecological processes or proxies: grazing, predation, recruitment and mean body size. Each process or proxy was modelled separately. Heterogeneity facet and organismal group were included as covariates.

### (g) Key environmental factors moderating heterogeneity effects (objective 2)

To assess the influence of environmental factors, we re-ran all facet (objective 1a) and organismal group (objective 1b) models with additional fixed effects: substrate type, depth, latitude and season. Substrate type was categorized as biogenic (macroalgae/fauna) or rock. Depth was classified as high/low intertidal, shallow (<15 m) or deep (>15 m) subtidal. Latitude was expressed in absolute decimal degrees, and the season standardized according to boreal seasons. Not all fixed effects were included in every model owing to data limitations (electronic supplementary material, S10). Where possible, interactions between season and latitude were tested, anticipating weaker seasonality effects at lower latitudes. Interactions between heterogeneity and substrate type were also tested, given that biogenic substrates (e.g. macroalgae) may inhibit certain processes (e.g. macroalgal sweeping effects on larvae settlement), while rock substrates may promote them.

### (h) Model validation and sensitivity analyses

Model assumptions were assessed using *Q–Q* plots and residual versus predicted plots [49]. Marginal and conditional *R*² values were used to quantify variance explained by fixed and random effects, respectively. Publication bias was evaluated by including sample size and publication year as fixed effects [50], with additional biases explored and reported (electronic supplementary material, S10–S12). Sensitivity analyses excluded large studies, HDRs with fewer than four data points, discrete heterogeneity variables, multi-facet HDRs and studies confounding heterogeneity with site, date or substrate (electronic supplementary material, S12). To ensure transparency and robustness, model structures were pre-specified, covariate testing was applied consistently, and sample sizes, effect sizes, confidence intervals and sensitivity results were fully reported ([51]; electronic supplementary material, S11 and S12).

Model outputs were used to assess the strength and nature of heterogeneity effects. Linear (*x*) and quadratic (*x*²) terms tested for the presence of heterogeneity effects and quantified the strength of heterogeneity relationships. Marginal *R*² quantified variance explained by fixed effects, while conditional *R*² reflected total variance explained, including random effects accounting for between-HDR and between-study variation.

## 3. Results

### (a) Heterogeneity effects across facets for richness and abundance in aggregated micro- and macroinvertebrate communities

**Figure 2.**
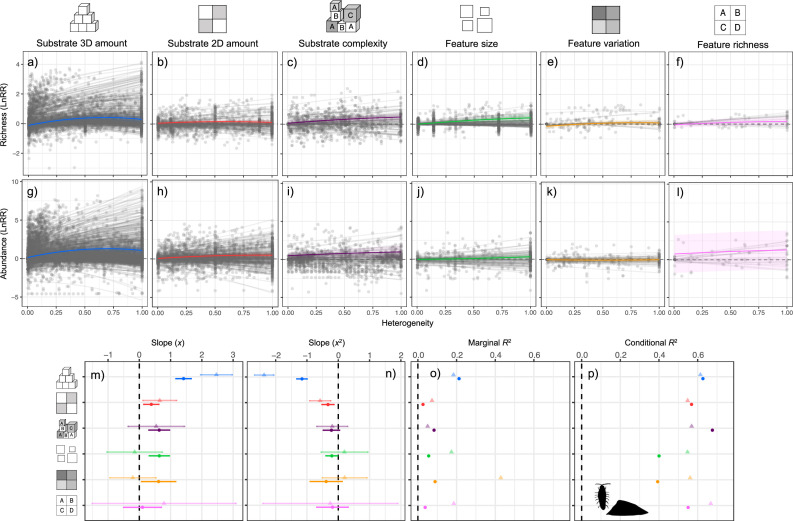
Effects of heterogeneity on species richness and abundance across heterogeneity facets. Results from mixed-effects linear models are shown as log response ratio (lnRR) effect sizes for species richness and abundance in the aggregated micro- and macroinvertebrate community data across heterogeneity facets. (a–f) Effects of heterogeneity (mean ± 95% CI) on species richness; (g–l) effects on abundance. Grey points represent observations, and grey lines indicate heterogeneity–diversity relationships (HDRs). The lower panels summarize: (m) linear effects (*x*; mean ± 95% CI), (n) quadratic effects (*x*²; mean ± 95% CI), (o) marginal *R*², and (p) conditional *R*² for species richness (circles) and abundance (triangles). A positive lnRR indicates an increase in richness or abundance with greater heterogeneity, while a negative lnRR suggests a decrease. The number of studies, number of HDRs and total number of observations included in each model are listed in electronic supplementary material, S13.

Heterogeneity promoted community richness and abundance in aggregated micro- and macroinvertebrate communities, following either a saturating or hump-shaped response (figure 2a–i), but the response varied across facets (figure 2m,n). For richness, five of the six facets showed positive HDRs, with richness most strongly increasing with substrate 3D amount (*t_x_*_,239_ = 11.29, *p* < 0.001; *$t_{x,3550}^{2}$* = −13.70, *p* < 0.001; figure 2a), and less strongly increasing with substrate 2D amount (*t_x,_*_208_ = 3.15, *p* = 0.001; $t_{x,1544}^{2}$ = −3.37, *p* < 0.001; figure 2b), substrate complexity (*t_x_*_,528_ = 3.77, *p* < 0.001; $t_{x,1017}^{2}$ = −1.65, *p* = 0.098; figure 2c), feature size (*t_x_*_,25_ = 3.89, *p* < 0.001; $t_{x,1237}^{2}$ = −2.15, *p* = 0.031; figure 2d) and feature variability (*t_x,_*_150_ = 2.20, *p* < 0.028; $t_{x,154}^{2}$ = −1.47, *p* = 0.141; figure 2e), respectively. Only two of the six heterogeneity facets showed positive abundance HDRs: abundance increased strongly with 3D amount (*t_x_*_*,*217_ = 9.47, *p* < 0.001; $t_{x,4817}^{2}$ = −14.95, *p* < 0.001; figure 2g) and less strongly with 2D amount (*t_x_*_,217_ = 2.37, *p* = 0.018; $t_{x,2265}^{2}$ = −3.35, *p* < 0.001; figure 2h). Neither richness nor abundance responded to increases in feature richness (figure 2f–l). Substrate 3D amount showed the most rapid increase, and the most pronounced saturation and humped shape, as indicated by the quadratic term in both richness and abundance models (figure 2n). For 3D amount, the abundance response substantially exceeded the richness response (figure 2m,n). Across all facets, confidence intervals were greater for abundance than for richness (figure 2m,n), with marginal *R*^2^ being the largest for the model including substrate 3D amount, but conditional *R*^2^ remaining relatively consistent across facets (figure 2o,p).

### (b) Heterogeneity effects on community responses across organismal groups

**Figure 3.**
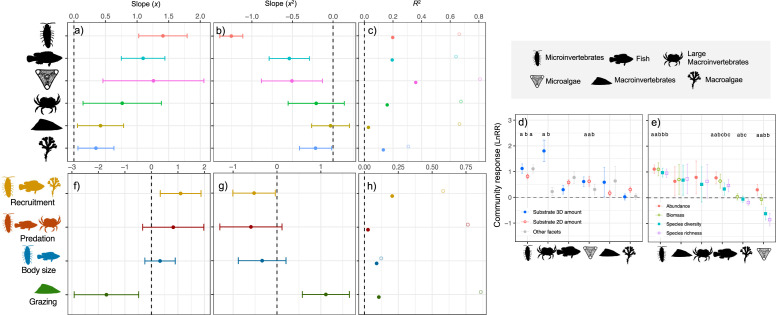
Heterogeneity effects on community responses and ecological processes across organismal groups. Results from mixed-effects linear term models are shown as lnRR effect sizes for community responses (diversity, abundance and biomass) across organismal groups (a–e) and for ecological processes (f–h). (a) Linear term (*x*; mean ± 95% CI), (b) quadratic term (*x*²; mean ± 95% CI), and (c) marginal (solid) and conditional (shaded) *R*² for organismal groups, combining abundance, biomass, richness and diversity. (d,e) Heterogeneity effects (mean ± 95% CI) on organismal groups across heterogeneity facets and response variables, with *post hoc* differences indicated by letters. (f) Linear terms and (g) quadratic terms, and (h) marginal and conditional *R*² for ecological process indicators. The number of studies, number of heterogeneity–diversity relationships and total number of observations included in each model are listed in electronic supplementary material, S8, S9 and S13.

Across organismal groups, microinvertebrates (*t_x_*_,164_ = 7.21, *p* < 0.001; $t_{x,12\;570}^{2}$ = −17.32, *p* < 0.001; figure 3a,b) and fish (*t_x_*_,150_ = 6.22, *p* < 0.001; $t_{x,1499}^{2}$ = −4.25, *p* < 0.001) presented the strongest community response to heterogeneity. Large macroinvertebrates (*t_x_*_,17_ = 2.36, *p* = 0.029; $t_{x,1260}^{2}$ = −1.22, *p* = 0.221), macroinvertebrates (*t_x_*_,74_ = 2.26, *p* = 0.026; $t_{x,2584}^{2}$ = −0.26, *p* = 0.792) and macroalgae (*t_x_*_,16_ = 2.39, *p* = 0.028; $t_{x,847}^{2}$ = −2.13, *p* = 0.033) presented the weakest responses. Microalgae (*t_x_*_,8_ = 3.08, *p* = 0.013; $t_{x,219}^{2}$ = −2.64, *p* = 0.008) presented moderately positive responses, but showed large variability. Microinvertebrates showed the most pronounced saturating or humped response (figure 3b). Marginal *R*^2^ fluctuated around 0.2 across the models for organismal groups, while conditional *R*^2^ values were just above 0.6 overall (figure 3c).

Differences across organismal groups remained largely consistent between heterogeneity facets and community response metric types (figure 3c). Significant differences across facets, with heterogeneity effects from substrate 3D amount being the largest, were consistent with the above results (figure 2m). Abundance responses, and sometimes biomass responses, were significantly larger than richness and diversity responses across various organismal groups (i.e. microinvertebrates, macroalgae, fish and microalgae; figure 3d). This, in turn, was again similar to the observed larger abundance effects across heterogeneity facets (figure 2m).

### (c) Heterogeneity effects across ecological process indicators

Heterogeneity clearly affected grazing and recruitment and had no clear effect on predation and body size (figure 3f,g), but note the limited sample size for grazing, predation and body size models (electronic supplementary material, S13). Grazing showed a negative, non-linear, response to heterogeneity (*t_x_*_,5_ = −2.73, *p* = 0.036; $t_{x,119}^{2}$ = 4.07, *p* < 0.001), although these studies exclusively addressed macroinvertebrate grazing. Recruitment responded positively to heterogeneity (*t_x_*_,17_ = 2.809, *p* = 0.011; $t_{x,649}^{2}$ = −2.11, *p* = 0.034). No significant effects were found for predation (*t_x_*_,59_ = 1.51, *p* = 0.135; $t_{x,104}^{2}$ = −1.63, *p* = 0.105; figure 3e,f) or body size (*t_x_*_,392_ = 1.11, *p* = 0.266; $t_{x,411}^{2}$ = −1.22, *p* = 0.223). Marginal *R*^2^ values for these models were around 0.1, while conditional *R*^2^ fluctuated a lot across models from 0.1 to almost 0.8 (figure 3h).

### (d) Biogeographical and environmental moderation of heterogeneity effects

**Figure 4.**
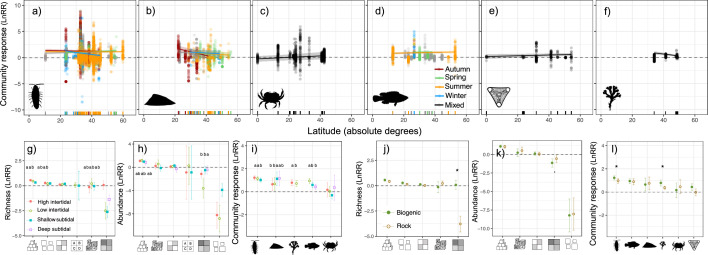
Influence of biogeographical and external environmental context on heterogeneity facets and organismal groups. Results from mixed-effects linear models are shown as lnRR effect sizes for community responses (diversity, abundance and biomass). (a–f) Responses across latitudes for different organismal groups: (a) microinvertebrates, (b) macroinvertebrates, (c) large macroinvertebrates, (d) fish, (e) microalgae and (f) macroalgae. (g–i) Depth effects on species richness and abundance across heterogeneity facets and organismal groups; (j–l) substrate effects. Lines show predicted trends, with 95% CI shaded or shown as error bars. Significant differences (*α* < 0.05) are marked with an asterisk (*), and *post hoc* differences in (g–l) are denoted by letters. A positive lnRR indicates an increase in response (e.g. richness, abundance) under greater heterogeneity, while a negative lnRR suggests a decrease. In (j,k), feature richness was excluded owing to a small dataset (j), and only presenting biogenic substrates (k). The number of studies, number of heterogeneity–diversity relationships and total number of observations included in each model are listed in electronic supplementary material, S13.

Latitude effects were intertwined with the season and varied across organismal groups. Microinvertebrates and macroinvertebrates responded more strongly to heterogeneity at lower latitudes, particularly in winter and autumn, respectively (latitude x season: *F*_3960_ = 6.98, *p* < 0.001; *F*_373_ = 4.46, *p* = 0.001; figure 4a,b). No patterns were found for large macroinvertebrates (latitude: *F*_79_ = 1.03, *p* = 0.305; figure 4c), fish (latitude: *F*_140_ = 1.34, *p* = 0.180; figure 4d) and microalgae (latitude: *F*_90_ = 1.06, *p* = 0.287; figure 4e). Although macroalgae showed a significant response (latitude: *F*_336_ = −2.30, *p* = 0.021; figure 4f), there was limited latitudinal data coverage in this case.

Differences between the intertidal and subtidal systems occurred across heterogeneity facets and organismal groups (figure 4g–j). Amongst the six significant comparisons, five showed stronger positive effects in the intertidal than in the subtidal (figure 4g–i). For macroinvertebrates, however, heterogeneity had the clearest positive effects in the subtidal (figure 4i).

Differences in heterogeneity effects across substrates showed significant effects across several models, with biogenic substrates showing a stronger positive community response for microinvertebrates, for macroalgae and for the aggregated micro- and macroinvertebrate richness responding to feature variation (figure 4j–l).

## 4. Discussion

**Figure 5.**
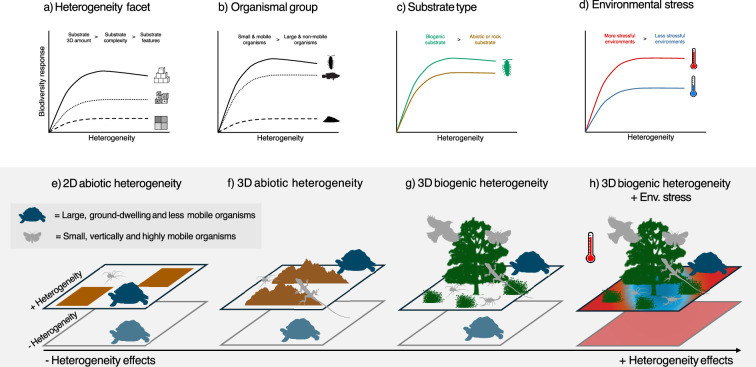
Hypothesized context-dependency of heterogeneity–diversity relationships on rocky reefs and across ecosystems. Heterogeneity effects on rocky reefs ecosystems were observed to be generally positive, saturating or hump-shaped, and varied depending on: (a) heterogeneity facets for well studied benthic invertebrates, (b) organismal groups and their associated traits (e.g. body size and vertical mobility), (c) substrate type (biogenic versus abiotic) and (d) environmental stress around temperature and desiccation (e.g. high intertidal zones). In this conceptual model, we hypothesize that heterogeneity facets, organismal traits and environmental stress more generally determine heterogeneity effects across both terrestrial and aquatic ecosystems. Specifically, we propose that heterogeneity effects will vary with: (e,f) facet, as 3D structures create broader and more finely structured environmental variation, (f,g) substrate type, as biogenic sources tend to produce more complex 3D structures than abiotic ones, and (g,h) environmental stress, where heterogeneity-provided refugia become more critical for sustaining biodiversity.

This study elucidates key sources of context-dependency in HDRs on rocky reefs, with potential application across systems. Although generally positive and showing linear, saturating and weakly hump-shaped forms, heterogeneity relationships were maximized when increasing the amount of 3D substrate and influencing microinvertebrates. In contrast, substrate features (e.g. variation in feature dimensions and richness), and responses of larger-bodied macroinvertebrates and macroalgae yielded the weakest effects, not being significant in some cases. Heterogeneity’s influence also tended to increase towards lower latitudes, in the intertidal, and with biogenic substrates. Together, these results underline the context-dependency of HDRs and suggest that these fundamental relationships (and heterogeneity’s predictive capacity) are determined by specific facets, organismal traits and environmental conditions. In particular, 3D structural elements for mobile species and in more stressful environments have the strongest influences and provide attractive opportunities for promoting biodiversity at the local scale (figure 5).

The strong effects of 3D structure likely arise from its simultaneous increase in substrate availability, environmental variability and vertical structure (figure 5a). Increased substrate area, especially for small-bodied organisms on topographically complex surfaces or habitat-forming species, enhances biodiversity through increased sampling and the provision of diverse habitat features. Importantly, the added vertical dimension from the benthos into the water column incorporates sharp environmental gradients (e.g. light, flow, temperature), creating varied niches and reducing stress (e.g. from desiccation or predation). Although it remains challenging to separate the effects of increased substrate from environmental variability [4,32], our analysis indicates that 3D structure is a powerful driver of biodiversity on rocky reefs at the local scale. By distinguishing different heterogeneity facets, HDRs can be interpreted through specific aspects, such as feature richness or variability, which allow researchers to investigate heterogeneity effects *per se*. However, facets like feature richness or variability alone may have weaker or more context-dependent effects, as they lack the structural complexity and environmental gradients of full 3D heterogeneity. Our novel comprehensive assessment allows comparisons of heterogeneity effects across a wide range of facets. The strong influence of 3D heterogeneity is consistent with prior syntheses in terrestrial systems, which show topographic and vegetative sources (both 3D) as major drivers [5], and extensive literature on 3D habitat-forming species [52,53]. Accordingly, we suggest that 3D heterogeneity is likely to generate the strongest biodiversity effects across ecosystems (figure 5e,f).

Heterogeneity effects varied across organismal groups, likely influenced by locomotion style and body size. Microinvertebrates benefited most, followed by fish, likely because both groups can climb, crawl or swim, allowing them to exploit 3D features like macroalgal canopies or boulders for food and predation refugia. The small size of microinvertebrates likely amplifies these benefits, providing better access to refugia, small-scale environmental variability and additional substrate area, as well as enabling specialized associations with specific substrates [54,55]. Microalgae also showed strong benefits, possibly owing to reduced macroinvertebrate grazing. Conversely, macroinvertebrates and macroalgae benefited least, potentially owing to structural elements that restricted macroinvertebrate movement, limited light for macroalgae, or hindered larval and spore settlement [56]. Similar patterns are expected in terrestrial systems, where 3D structures may favour small-bodied invertebrates and vertically mobile organisms like climbing mammals, reptiles or birds [57]. Overall, these findings extend our understanding of heterogeneity effects across trophic and taxonomic groups [1,5], suggesting that key traits likely determine organisms' ability to exploit heterogeneous environments and the strength of their response (figure 5b).

Biogenic 3D substrates provided larger heterogeneity effects—particularly on microinvertebrates—presumably by generating larger and more intricately varied heterogeneity structures and allowing more obligate relationships with species [54,55], including feeding interactions. Biogenic substrates may have also promoted macroalgae by hampering the movement and settlement of macroinvertebrates by generating structures that prevented their access to small spaces and would have otherwise reduced the settlement of larvae or algal propagules on the rock [58,59]. Importantly, 3D substrate amount exerted stronger effects on communities than other facets regardless of whether biotically or abiotically generated, underlining the primacy of the heterogeneity facet in driving outcomes. Nevertheless, accounting for substrate type provides additional capacity to explain heterogeneity responses on rocky reefs, and given the ability of biogenic structures such as mosses, shrubs, corals and sponges to engineer highly heterogeneous environments, we expect biogenic heterogeneity to have greater potential to boost biodiversity across ecosystems (figure 5c,f,g).

Heterogeneity effects varied across depths and latitudes, suggesting a possible influence of environmental stress. Effects on the best-studied groups—microinvertebrates and macroinvertebrates—were generally stronger in intertidal zones, where intense thermal and desiccation stress supports the hypothesis that biodiversity benefits more from heterogeneity-driven stress relief under extreme conditions [60–62]. Particularly in the intertidal zone, stronger heterogeneity effects towards the tropics may similarly reflect an intensification of environmental stress [63,64], although latitude—stress relationships are complex in intertidal systems [60]. Larger heterogeneity effects near the tropics may also reflect a greater species pool ([65]; but see [66]), along with lower seasonality or higher productivity [67], as well as stronger predation [68]. Data from tropical regions, however, were limited, possibly owing to the prevalence of coral reefs over rocky reefs—despite large rocky reef systems in e.g. the Bahamas [69]. Other studies suggest that positive species interactions, particularly with habitat-forming species, become more critical with increased environmental stress [70]. To our knowledge, these findings are the first to show that heterogeneity effects may intensify with environmental stress on a global scale, highlighting an overlooked source of HDR variability (figure 5d) and underscoring the importance of managing and restoring heterogeneity in a world facing rising stressors.

Extending its role beyond diversity and abundance, heterogeneity also influenced multiple ecological processes, highlighting potential indirect effects on biodiversity. On rocky reefs, it promoted recruitment, likely owing to species associating with structures like rock pools or macroalgal canopies [35,71]. Large, heterogeneous structures often support recruitment [2,21] but can sometimes reduce post-settlement survival [72]. Our results also showed negative effects on grazing, which is unusual, as most terrestrial studies report the reverse, where grazing modifies heterogeneity (e.g. cattle altering shrublands [73]). Positive effects on grazing may also arise, such as sea urchins using rock crevices for refuge [28] or grazing fish benefiting from heterogeneity-driven algal growth [74]. Moreover, neither predation nor body size showed a clear response to heterogeneity, although limited data restrict firm conclusions. The lack of response in mean body size could be due to heterogeneity simultaneously promoting both small invertebrates and large fish. Likewise, variability in predation responses could stem from complex interactions among heterogeneity, predation and biodiversity [20,75]. Nevertheless, these findings indicate that heterogeneity’s effects on grazing and recruitment likely underpin diversity responses and can therefore be essential in restoring degraded habitats, highlighting heterogeneity as a key factor in restoration projects [76–79].

Across facets, organisms and ecological processes, heterogeneity effects varied in shape and were largely—but not all—positive. The majority of the HDRs appeared to saturate at high levels of heterogeneity. Saturating HDRs are common across systems and communities, indicating a reduction in the number of niches or space available as heterogeneity increases, or that local richness is close to species pool richness [25,65]. Three cases in our study showed a slightly humped shape, which coincided with the largest effects for each group of models and were generated by the facet 3D amount. Humped HDRs have been linked to the creation of small, inaccessible spaces, with small populations vulnerable to stochastic effects [14,15]. It is also possible that the very highest levels of 3D structure led to diminished environmental heterogeneity, as dense structures dominate the environment and its conditions (e.g. very dense canopy reducing heterogeneity of light availability in the understorey). Our results indicate that, while heterogeneity generally supports greater abundance and diversity, we should expect diminishing returns and even biodiversity declines as the highest levels of 3D heterogeneity are reached.

Several limitations should be considered when interpreting our findings. First, we standardized heterogeneity data for comparability across metrics, making the units relative to the original studies and not applicable to new contexts. This may have also introduced some unexplained variability in our models. Second, although recent studies have raised concerns about using fractal indices to measure complexity [4,40], we included it owing to its historical use in ecology [29] and to allow comparisons with other metrics and facets. Third, metrics based on substrate area or volume within a constant basal area add a third dimension of heterogeneity that covaries with realized substrate area. This added dimension is likely one mechanism by which 3D structure enhances biodiversity (see second Discussion paragraph above). It remains relevant in biodiversity or ecosystem functioning studies that use units defined by constant planar area (e.g. productivity, typically measured as gC m^−2^ basal area yr^−1^) or in managing areas with limited planar footprints, such as marine protected areas or seawalls. Fourth, excluding the small number of studies that quantified multiple facets led to an increase in model-estimated heterogeneity effects (electronic supplementary material, S12). These studies were likely more exploratory, presented weaker effects, and thus led to a ‘dilution’ of heterogeneity effects. Finally, our dataset is biased towards 3D and 2D structures in temperate regions, with a focus on abundance and richness, but latitudinal patterns can also be found with limited tropical coverage [80,81]. There is limited research on other aspects of heterogeneity, such as feature richness, tropical regions, and ecosystem processes, which should be explored in future studies.

## 5. Conclusions

Our results reveal key factors in local heterogeneity effects on communities and ecological processes across marine rocky reefs worldwide. Community responses are strongest with heterogeneity generated by biogenic substrates and 3D structures, particularly where focal organisms have traits that enable optimal use of complex habitats and in environmentally stressful conditions. We propose a coherent set of facets as a framework to guide future syntheses and direct research toward understudied heterogeneity facets, organismal groups, ecological processes (electronic supplementary material, S14) and biogeographical regions. Finally, we offer general predictions for key sources of context-dependency in HDRs, aiming to encourage further testing across ecosystems.

## Data Availability

Data and code are available at Dryad [82]. Supplementary material is available online [83].
